# The value of histological examination in the diagnosis of tuberculous lymphadenitis in the era of rapid molecular diagnosis

**DOI:** 10.1038/s41598-022-12660-0

**Published:** 2022-05-27

**Authors:** Sabira Tahseen, Atiqa Ambreen, Sheeba Ishtiaq, Faisal M. Khanzada, Nauman Safdar, Lisbet Sviland, Tehmina Mustafa

**Affiliations:** 1 Department of Global Public Health and Primary Care, Centre for International Health, University of Bergen, Bergen, Norway; 2 National TB Reference Laboratory, National Tuberculosis Control Program Pakistan, Block E and F, EPI building, Near NIH, Chak Shahzad, Islamabad, Pakistan; 3 Department of Microbiology, Gulab Devi Hospital, Lahore, Pakistan; 4 Department of Pathology, Gulab Devi Hospital, Lahore, Pakistan; 5 Social and Health Inequalities Network (SHINe) Pakistan, Islamabad, Pakistan; 6 Department of Clinical Medicine, Faculty of Medicine, University of Bergen, Bergen, Norway; 7 Department of Pathology, Haukeland University Hospital, Bergen, Norway; 8 Department of Thoracic Medicine, Haukeland University Hospital, Bergen, Norway

**Keywords:** Microbiology, Molecular biology, Diseases, Health care, Medical research, Signs and symptoms

## Abstract

Extrapulmonary tuberculosis often poses a diagnostic challenge. This study aimed to assess the value of histological examination in diagnosing tuberculous lymphadenitis (LNTB) when performed simultaneously with rapid molecular assay (Xpert MTB/RIF) testing. People presumed to have LNTB were prospectively enrolled in a tertiary care hospital. Excision biopsy was performed and tested by histology, Xpert, and culture. Of 390 lymph nodes, 11 (2.8%) were positive by AFB microscopy, 124 (31.8%) by Xpert, 137 (35.1%) by culture, and histopathology was consistent with TB in 208 (53.3%). Altogether, LNTB was diagnosed in 228 and bacteriologically confirmed TB in 178 cases. Against culture, histopathology versus Xpert had higher sensitivity (93 vs. 62%) but lower specificity (68 vs. 83%). In patients with short clinical history, a significantly higher number of Xpert-positive specimens were culture-positive. Among patients with histology suggestive of TB, no difference was seen in response to treatment between bacteriology positive and negative, but a significant slow response was noted in bacteriology confirmed TB with nonspecific histology. In a country like Pakistan, with high TB and low HIV prevalence, diagnosis is possible for more than 95% of LNTB when Xpert and histopathology examination is used in combination, compared to less than 60% by Xpert alone.

## Introduction

Every year 10 million people fall ill with tuberculosis (TB). TB is caused by *Mycobacterium tuberculosis* (MTB) which primarily affects the lungs causing pulmonary TB (PTB) but can also affect other organs, causing extrapulmonary tuberculosis (EPTB). In 2020 EPTB accounted for 18% of 5.8 M TB cases notified globally^1^. Commonest disease manifestations of EPTB include lymph nodes, pleura, abdominal, and osteoarticular system^2^. Patients with EPTB often have constitutional symptoms, and specific symptoms based on the tissue or organ affected^2,3^. The diagnosis of EPTB often poses challenges either because of its occult nature, difficulty in obtaining samples, or the paucibacillary nature of the disease^3^. Specimen from the extrapulmonary disease site is usually obtained by aspiration or biopsy, and the laboratory diagnosis of tuberculosis is made by histological and bacteriological examination^2–4^. Histologic diagnosis is based on identifying granulomatous inflammation commonly characterized by aggregates of epithelioid histiocytes, with a peripheral cuff of lymphocytes and plasma cells. The epithelioid cell may also coalesce to form multinucleated giant cells, and central necrosis is distinctly present in necrotizing granulomas. *Mycobacteria* species are the most common etiologies of necrotizing granulomas^4,5^. The sensitivity of histological features has been reported to vary from 59 to 88% for lymph node TB (excisional biopsy) against a composite reference standard^6^. The histologic examination has limitations in the diagnosis of TB; the granulomatous response may fail to elicit in immunocompromised individuals with TB; furthermore, granulomatous responses are also seen in infectious other than tuberculosis, autoimmune, toxic, allergic, and neoplastic conditions^4–6^. For bacteriological diagnosis of EPTB, acid-fast bacilli (AFB) microscopy has a limited value because of its low sensitivity. The culture is regarded as the gold standard for the diagnosis of TB; still, its use is limited by the requirements of sophisticated laboratory infrastructure, freshly collected specimens to ensure bacilli’s viability, and a long turnaround time^2,6^. Molecular methods for the diagnosis of TB have evolved over the last two decades^7,8^. The first automated real-time nucleic acid amplification (NAA) technology for rapid and simultaneous detection of tuberculosis and rifampicin resistance, Xpert MTB/RIF® assay (Cepheid, Sunnyvale, CA, USA), was endorsed by WHO in 2010^9^. The Xpert MTB/RIF assay(Xpert) is sensitive and more rapid than conventional methods, with higher feasibility for implementation close to the point of care because of minimal infrastructure requirement^7,8^. The policy recommendations have evolved with increasing use and evidence^10–13^. In 2013, WHO, for the very first time, recommended the use of Xpert for the diagnosis of TB in selected extrapulmonary specimens, including lymph nodes^14^. In 2017, WHO endorsed the next-generation Xpert MTB/RIF ultra ® assay with improved detection limit and sensitivity similar to solid culture^15^. In light of additional evidence, in 2021, WHO updated its guidelines and recommended using Xpert and Xpert Ultra as initial diagnostic tests rather than AFB microscopy and or culture for pulmonary and extrapulmonary specimens^16–18^.

International Standard for Tuberculosis care (ISTC) for a patient with presumed EPTB recommends obtaining appropriate specimens for microbiology testing and histologic examination^19,20^. The laboratory diagnosis of EPTB in high TB burden countries relied on clinical judgment and identifying granulomatous inflammation in biopsied samples rather than bacteriological examination because of the lack of appropriate diagnostic facilities^2^. With the advancement in molecular technology, access to rapid and more sensitive diagnostic tools for TB has dramatically improved. Tuberculosis disease cannot be ruled out with a negative smear and NAA test result. For this reason, histopathology examination and culture are used in conjunction with more rapid AFB smear and NAA tests in developed countries^6,7^. However, clear guidance is lacking on the use of histologic examination to diagnose TB. We conducted this study to evaluate the added value of histological examination in the diagnosis of TB in patients presumed to have TB lymphadenitis by comparing histologic and molecular diagnosis using culture as the gold standard. We also evaluated the difference in clinical outcomes of LNTB patients diagnosed by bacteriology and/or histologic features.

## Study setting, population, and methodology

This study was part of a larger research project aimed at improving the diagnosis of EPTB under routine programmatic conditions in Pakistan^21,22^.

### Study setting

Pakistan is a high TB and low HIV prevalent country^1^. The study was conducted in a private, not-for-profit tertiary care hospital located in the capital city of the largest province. The hospital specializes in TB care services; presumptive and already diagnosed TB patients are referred to Gulab Devi Hospital (GDH) for consultation and second opinion. Annually, almost 6000 TB patients are registered for TB treatment at GDH, and many others, after the diagnosis, are referred back for treatment to TB clinics closer to their residence. Routinely, all patients presumed to have EPTB are investigated for concomitant PTB by sputum AFB smear microscopy examination. For presumptive TB lymphadenitis, an excision biopsy is performed for diagnosis. The hospital has laboratory facilities for histopathology and mycobacteriology, including AFB microscopy, Xpert MTB/Rif, solid and liquid culture, and *M. Tuberculosis* isolates are sent to National TB Reference Laboratory Islamabad for drug susceptibility testing (DST)^21^. Laboratory reports are issued routinely within 24–48 h for AFB smear and Xpert and within 4–6 days for a histopathology examination.

### Study population and methodology

Patients of all ages and gender presenting in out-patient clinics with enlarged lymph nodes presumed to have LNTB were evaluated for inclusion. Patients with a history of previous TB treatment and/or an already established diagnosis of LNTB were excluded. Eligible patients were enrolled prospectively after informed consent.

All enrolled patients were asked to submit sputum samples. An excision biopsy of the lymph nodes was performed. Among patients diagnosed with LNTB, those who opted for treatment at GDH were interviewed using a structured questionnaire and followed till completion of the first-line anti TB treatment (FL-ATT) as described previously^22^. Chest X-rays available in files of the enrolled patient were reread for the evidence of intrathoracic lymph nodes, PTB, and any other abnormality.

### Laboratory methods

The LN biopsy specimen, immediately after excision, was divided into two; one half was placed in 10 ml physiologic saline (0.9%), and the other half was placed in buffered formalin and then transferred to the laboratory.

The LN biopsy specimen in saline was processed for AFB smear, Xpert, and culture for bacteriology examination. The biopsy specimen in saline was first minced in a manual sterile tissue homogenizer (15 ml; 19 × 155 mm), then transferred to a sterile 50 ml tube, processed without decontamination, and then centrifuged (3000 g for 15 min). The sediment was used for smear, Xpert, and culture examination^21,23^. AFB smears were stained with Auramine-O and examined using a light-emitting diode(LED) fluorescence microscope^24^. Xpert was performed using manufacturer protocols^25^. For culture, two slopes of Lowenstein-Jensen (LJ) medium and one Mycobacteria Growth Indicator Tube (MGIT 960; Becton Dickinson, Sparks, MD, USA) were inoculated^23^.

The LN biopsy specimen in buffered formalin was processed for histopathology examination, and sections were stained with hematoxylin and eosin (H&E); a histopathologist at GDH examined the sections. For this study, the H&E stained slides of all LNs reported having morphological features consistent with TB were reexamined. Histopathology reports were structured into four groups (i) well-formed granulomas with no necrosis. (ii) well-formed granulomas (predominantly)with necrosis (iii) ill-formed granulomas with necrosis (iv) caseous necrosis with no granuloma^26^.

The sputum samples were examined by AFB microscopy only.

### Definitions

Histopathology consistent with TB was defined as morphological features showing well or poorly formed granuloma and/or caseous necrosis with or without Langhans type giant cell. Bacteriology confirmed LNTB (B + ve LNTB) was defined as MTB positive by Xpert and/or culture. The composite reference standard (CRS) for TB was defined as MTB positive by Xpert and/or culture and/or histopathology consistent with TB.

Duration of illness was calculated from the first symptom, and a cut-off of three months was used to stratify short from long periods of illness. Successful treatment outcome was defined as clinical response with standard of care when treatment was given for six months and extended treatment if given for more than six months. Patients whose clinical response was documented in the treatment card at the end of five months but who didn’t come for the last follow-up were also counted as successfully treated with standard treatment.

### Data management and statistical analysis

Data was entered in EpiData Manager v4.2 (Epi-Data Association, Odense, Denmark) and analyzed using Stata v13 (Stata Corporation, College Station, TX, USA). Data on demography, clinical symptoms, duration of illness, treatment duration, and the outcome was extracted from the questionnaire and patient treatment card. Only cases with interpretable histology and bacteriology examination results were analyzed, and cases with tissue other than lymph nodes or neoplastic disease on histological examination were excluded from the analysis.

Categorical variables were reported as frequencies and percentages. The performance of the Xpert and histology was calculated against culture as a microbiological reference standard. Cross-tabulation was used to calculate sensitivity (Se), specificity (Sp), positive predictive value (PPV), and negative predictive value (NPV). Two sample/group proportion tests were used to compare the response to treatment and analyzed using binary logistic regression to calculate the odds ratio (OR) and 95% confidence interval (95% CI). *P* value < 0.05 was considered statistically significant.

### Ethical considerations

The study protocol was approved by the National Bioethics Committee of Pakistan (Islamabad, Pakistan) and the Regional Committee for Medical and Health Research Ethics, Western-Norway (REK Vest). University of Bergen (Postboks 7804, 5020 Bergen, Norway). Eligible study participants were enrolled after informed consent. The de-identified data was used for analysis. The design and reporting of the study followed the guidelines for reporting diagnostic accuracy studies (2015).

## Results

**Figure 1:**
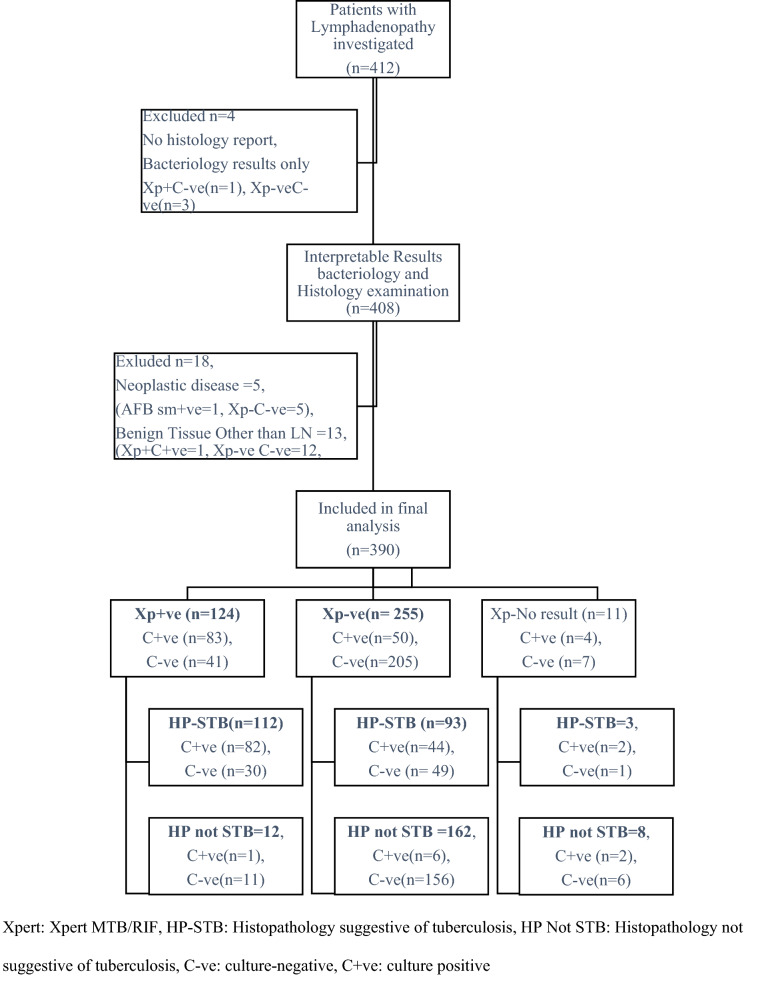
Flow diagram showing people investigated for enlarged lymph nodes presumed to have TB and the rapid molecular assay, histology, and culture examination results.

Altogether 412 patients with enlarged lymph nodes were enrolled from April 2016 to August 2017. Twenty-two cases, including four, reported having no tissue, 13 with tissue other than lymph nodes, and five with the neoplastic disease were excluded (Figure 1). Among 390 patients included, AFB microscopy was positive in 11 (2.8%), Xpert detected MTB in 124 (31.8%), culture was positive in 137 (35.1%), and histopathology was consistent with TB in 208 (53.3%). LNTB was diagnosed in 228 (58.5%) based on CRS, and bacteriology confirmed LNTB in 178 (45.6%) cases.

### Demography and clinical features

Demographic features are shown against the diagnostic profile of LNTB patients in Table 1. The median age was 19 yrs, and 61% were females. All patients presented with lymphadenopathy; 12 (5%) patients also had abscess formation, and 7 (3%) had discharging sinuses. Of patients diagnosed with LNTB (n = 228), HIV status was known for one HIV reactive and 71 non-reactive patients. Among LNTB patients, 183 opted for treatment at GDH, 147 consented to in-depth interviews, and chest X-rays were available in the files of 153 patients. Among LNTB patients, 38% (n = 87) were sick for 3 months or less and 35% (n = 80) for more than 3 months, and history was not recorded by the remaining 27% (n = 61). Of the 147 LNTB patients interviewed, symptom reported included fever (89%), current cough (52%), weight loss (37%), appetite loss (30%) and night sweat (14%). Among 153 chest X-rays reread, 30% reported intrathoracic lymph nodes, 10% had abnormal shadows suggestive of PTB, two showed pleural effusion, and three had other abnormalities, whereas, in 54% (n = 83), no abnormality was seen (Table 1).

**Table 1:** Demographic characteristics and clinical features of lymph node tuberculosis patients and results of histopathology and bacteriology examination.

	All (n)	Morphological features on histology examination					Bacteriology results			
		HP-0 n (%)	HP-1 n (%)	HP-2 n (%)	HP-3 n (%)	Hp-4 n (%)	Xp + ve C + ve n (%)	C + ve (only) n (%)	Xp + ve (only) n (%)	XP−ve C−ve n (%)
All LNTB	228	20 (9%)	4 (2%)	109 (48%)	82 (36%)	13 (6%)	83 (36%)	54 (24%)	41 (18%)	50 (22%)
Median age (yrs.)	19	15	31.5	19	19.5	25	19	19	19.5	19
Female	140	12 (9%)	4 (3%)	71 (51%)	45 (32%)	8 (6%)	48 (34%)	36 (26%)	26 (19%)	30 (21%)
**Age-Group (yrs.)**										
< 5 yrs	6	3 (50%)	0	1 (17%)	2 (33%)	0%	1 (17%)	1 (17%)	3 (50%)	1 (17%)
5–14 yrs	58	7 (12%)	0	32 (55%)	16 (28%)	3 (5%)	18 (31%)	7 (12%)	7 (12%)	26 (45%)
> 15 yrs	164	10 (6%)	4 (2%)	76 (46%)	64 (39%)	10 (6%)	64 (39%)	33 (20%)	31 (19%)	36 (22%)
**Duration of illness**										
< 3 months	90	5 (6%)	2 (2%)	40 (44%)	36 (40%)	7 (8%)	45 (50%)	14 (16%)	10 (11%)	21 (23%)
> 3 months	77	3 (4%)	1 (1%)	50 (65%)	19 (25%)	4 (5%)	22 (29%)	22 (29%)	14 (18%)	19 (25%)
NA	61	12 (20%)	1 (2%)	19 (31%)	27 (44%)	2 (3%)	16 (26%)	18 (30%	17 (28%)	10 (16%)
**HIV status**										
Positive	1	0	0	1 (100%)	0	0	0	1 (100%)	0	0
Non-Reactive	70	6 (9%)	1 (1%)	29 (41%)	31 (44%)	3 (4%)	19 (27%)	22 (31%)	10 (14%)	19 (27%)
Unknown	157	14 (9%)	3 (2%)	79 (50%)	51 (32%)	10 (6%)	64 (41%)	31 (20%)	31 (20%)	31 (20%)
**Local Symptoms**										
LAP only	209	20 (10%)	4 (2%)	102 (49%)	74 (35%)	9 (4%)	74 (35%)	52 (25%)	38 (18%)	45 (22%)
LAP and abscess	12	0	0	3 (25%)	6 (50%)	3 (25%)	8 (67%)	2 (17%)	1 (8%)	1 (8%)
LAP and sinus	7	0	0	4 (57%)	2 (29%)	1 (14%)	1 (14%)	0	2 (29%)	4 (57%)
**Constitutional Symptoms**										
Patient interviewed	147	6 (4%)	3 (2%)	77 (52%)	52 (35%)	9 (6%)	56 (38%)	33 (22%)	18 (12%)	40 (27%)
Fever	131	6 (5%)	2 (2%)	66 (50%)	49 (37%)	8 (6%)	50 (38%)	30 (23%)	17 (13%)	34 (26%)
Cough	76	3 (4%)	2 (3%)	37 (49%)	29 (38%)	5 (7%)	25 (38%)	23 (30%)	12 (16%)	16 (21%)
Night sweats	21	1 (5%)	0	9 (43%)	9 (43%)	2 (10%)	8 (38%)	6 (29%)	2 (10%)	5 (24%)
Weight loss	55	1 (2%)	0	24 (44%)	25 (45%)	5 (9%)	21 (38%)	14 (25%)	5 (9%)	15 (27%)
Appetite loss	44	1 (2%)	0	24 (55%)	14 (32%)	5 (11%)	18 (41%)	11 (25%)	4 (9%)	11 (25%)
**Chest X-Ray findings**										
X-Ray Reread	153	5 (3%)	3 (2%)	86 (56%)	50 (33%)	9 (6%)	59 (39%)	34 (22%)	21 (14%)	39 (25%)
PTB-suggestive	14	1 (7%)	0	6 (43%)	5 (36%)	2 (14%)	4 (29%)	3 (21%)	4 (29%)	3 (21%)
Intra-thoracic LN	51	1 (2%)	0	34 (67%)	14 (27%)	2 (4%)	24 (47%)	12 (24%)	4 (8%)	11 (22%)
Pleural effusion	2	0	0	2 (100%)	0	0	1 (50%)	0	0	1 (50%)
Other abnormalities	3	0	0	3 (100%)	0	0	2 (66%)	0	0	1 (33%)
No abnormality	83	3 (4%)	3 (4%)	41 (49%)	31 (37%)	5 (6%)	28 (34%)	19 (23%)	13 (16%)	23 (28%)

HP-0: histological, not suggestive TB, HP-1: Well-formed granulomas no necrosis, HP-2: well-formed granuloma with necrosis, HP-3: ill-formed granulomas with necrosis, HP-4: Caseous necrosis only.

LAP: Lymphadenopathy, XP: Xpert MTB/RIF, C: culture, LN: Lymph node.

Xp + ve C + ve: MTB detected by Xpert MTB/RIF and grown on culture.

Xp + ve only: MTB detected by Xpert MTB/RIF and no growth on culture.

C + ve only: MTB not detected or results not interpretable, MTB grown on culture.

XP−ve C−ve: MTB-Not detected or results not available on Xpert MTB/RIF and no growth on culture.

### Laboratory results

#### AFB microscopy

**Figure 2:**
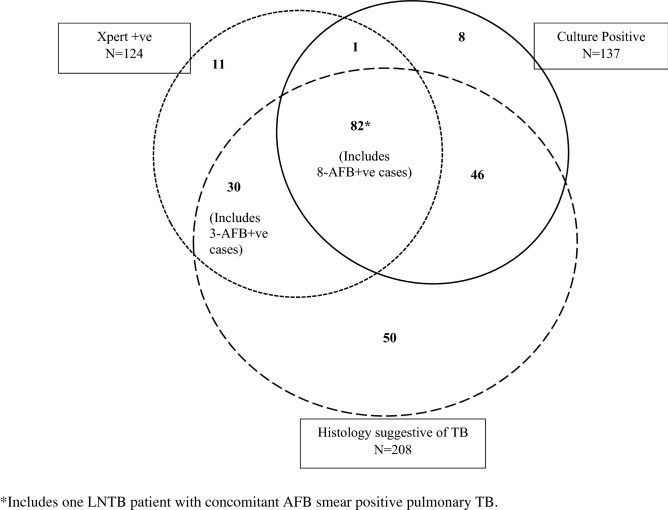
Venn diagram showing Xpert MTB/RIF, culture, and histology results of 228 tuberculous lymphadenitis patients diagnosed using composite reference standard.

Of 390 LN tissues examined, 11 were positive for AFB microscopy, and 64% were scant positive. All 11 AFB positive LN were positive for MTB by Xpert, and eight were culture positive (Figure 2). Sputum was examined from 224/228 LNTB patients; only one was reported AFB smear-positive.

#### Xpert MTB/RIF results and performance

Of 390 LN examined, interpretable Xpert results were reported in 379 (97%). MTB was detected in 124 LN, and in 93%, MTB detected was low or very low in quantity based on the cut-off threshold value (Table 2). Of 124 MTB positive, 66.9% (n = 83) were culture positive, ranging from 100% in high, 75% in medium, 70% in low, and 63% with a very low quantity of MTB. The Se, Sp, PPV, and NPV of molecular diagnosis against culture were 62%, 83%, 67%, and 80%, respectively. A significantly higher Se (*P* = 0.012) and PPV (*P* = 0.028) for Xpert was reported in subgroups of patients having short clinical history (3 month or less) compared to those ill for longer than three months at the time of presentation.

**Table 2:** Performance of Xpert MTB/Rif assay against culture in people with short (≤ 3 months) versus long clinical history (> 3 months).

Xpert MTB/RIF	Duration of illness /Culture results								
	Any duration of illness			< 3 Months		> 3 Month		No information	
	All n	Positive n	Negative n	Positive n	Negative n	Positive n	Negative n	Positive n	Negative n
All tested	390	137	253	59	44	44	47	34	162
Interpretable results (n)	379	133	246	59	44	42	46	32	156
**MTB-detected**									
High	1	1	0	1	0	0	0	0	0
Medium	8	6	2	2	0	3	0	1	2
Low	53	37	16	24	5	7	5	6	6
Very low	62	39	23	18	5	12	9	9	9
**MTB-detected-All**	124	83	41	45	10	22	14	16	17
**MTB not detected**	255	50	205	14	34	20	32	16	139
**Performance characteristics % (95%CI)**									
Sensitivity		62.4%	(54.3–70.4)	76.3%	(64.0–85.3)	52.4%	(37.7–66.6)	50.0%	(35.2–67.5)
Specificity		83.3%	(79.4–88.1)	77.3%	(63.0–87.1)	69.6%	(55.2–80.9)	89.1%	(84.6–93.7)
Positive predictive value		66.9%	(58.1–74.3)	81.8%	(69.7–89.8)	61.1%	(44.9–75.2)	48.5%	(33–64.4)
Negative predictive value		80.4%	(76.8–85.9)	70.8%	(56.8–81.8)	61.5%	(47.9–73.5)	89.7%	(85.2–94.1)

The difference in the performance of Xpert between subgroups of patients having short clinical history compared to those with a longer history of illness, sensitivity (*P* = 0.012), specificity (*P* = 0.409), PPV (*P* = 0.028), and NPV (*P* = 0.327).

#### Histological examination results and performance

Histopathology suggestive of TB was reported in 208 cases, including necrotizing granulomatous inflammation in 191 (92%) and caseous necrosis without granuloma in 13 (6%), and granulomatous inflammation without necrosis in four cases (2%) (Table 3).

**Table 3:** Performance of histological examination against culture, Xpert MTB/Rif assay, and bacteriology in the diagnosis of tuberculous lymphadenitis.

Histopathology	Bacteriology results						
	Total reported n	Culture		Xpert		Bacteriology	
		Positive n	Negative n	Positive n	Negative n	Positive* n	Negative n
Total reported	390	137	253	124	255	178	212
**Suggestive of TB**							
WFG no necrosis	4	2	2	2	2	4	0
WFG with necrosis	109	68	41	54	52	82	27
IFG with necrosis	82	50	32	45	37	61	21
C Necrosis (only)	13	8	5	11	2	11	2
**Suggestive of TB (all)**	208	128	80	112	93	158	50
**Not suggestive of TB**	182	9	173	12	162	20	162
**Performance characteristics %(95%CI)**							
Sensitivity		93%	(87.9–96.5)	90%	(83.9–94.4)	89%	(83.3–92.6)
Specificity		68%	(62.4–73.8)	64%	(57.5–69.2)	76%	(70.3–81.6)
Positive predictive value		62%	(54.8–67.9)	55%	(47.8–61.3)	76%	(69.7–81.3)
Negative predictive value		95%	(90.9–97.4)	93%	(88.3–96.0)	89%	(83.6–92.8)

HP: Histopathology, WFG: Well-formed granuloma, IFG: Ill-formed granuloma, C. Necrosis: Caseous necrosis, TB: tuberculosis.

*Bacteriology positive defined as positive on Xpert and/or Culture.

Among 208LN with histopathology suggestive of TB, 82(39%) were positive both by Xpert and culture, 30(14%) by Xpert only, 46(22%) by culture only, and 50 were negatives by both (Figure 2).

In Table 3, the performance of histological examination is shown against culture, Xpert, and B + LNTB. The Se, Sp, PPV and NPV for histological diagnosis against B + LNTB was 89%, 76%, 76%,and 89% respectively. Performance of histological examination was higher against culture compared to Xpert as the reference standard, but the difference was not statistically significant for Se (*P* = 0.384), Sp (*P* = 0.341), PPV (*P* = 0.149), and NPV (*P* = 0.422).

Histopathology was not consistent with TB in 20/178 B + LNTB cases. In these cases, reactive lymphoid hyperplasia was reported in 14, chronic nonspecific inflammation in four, acute suppurative inflammation, and lymphoproliferative disorder in one each.

Among Xpert positive, the culture positivity was 73.2%(95%CI; 64.3–80.6) in LN tissues showing TB specific morphological features, compared to 8.3% (95% CI;1.5–35.3) in tissues showing non-specific morphology (*P* < 0.001).

### Drug resistance

On phenotypic DST, rifampicin resistance was reported in two and mono resistance to isoniazid in six LNTB patients^21^. Of rifampicin-resistant, one case was negative and the other positive by AFB microscopy and Xpert. Rifampicin resistance was not detected by Xpert in both cases. Of six INH-resistant cases, all LNTB cases were AFB microscopy negative, and four were MTB positive by Xpert.

The necrotizing granulomatous lesion was reported histologically in all eight drug-resistant cases.

### Treatment outcomes

Of all LNTB patients diagnosed, 183 were registered for FL-ATT at GDH. Of these, 90% (n = 165) completed treatment, 9.3% (n = 17) were lost to follow up and one was declared treatment failure. Of 165 who completed treatment, 73% (n = 121) were treated successfully with a six-month standard treatment, and 27% (n = 45) after an extended treatment due to delayed clinical response. Predictors of slow treatment response have been reported previously^22^. Treatment outcomes and delayed treatment response in subgroups of TB patients stratified by diagnostic results are shown in Table 4. Two rifampicin-resistant and 5/6 INH-resistant LNTB patients were started on standard FL-ATT. One rifampicin-resistant TB patient was lost to follow up after two months, and the other completed treatment in 6 months. Of five INH-resistant LNTB patients, two completed treatment successfully in six months, whereas treatment was extended for 2, 3, and 6 months in the other three patients.

**Table 4:** Diagnostic results and treatment outcomes and duration of first-line TB treatment of Tuberculous lymphadenitis patients enrolled at Gulab Devi Hospital.

	LNTB patient diagnosed	Enrolled on Treatment	Treatment completed		Duration of treatment				OR	95%CI	*P* value
					6 months		> 6 months				
	n	n	n	%	n	%	n	%			
**All Patient**	228	183	165	90%	121	73%	44	27%			
**Gender**											
Male	88	69	60	87%	48	80%	12	20%	Ref		
Female	140	114	105	92%	73	70%	32	30%	1.75	0.78–4.1	0.14
**Age Group**											
< 15yrs	64	49	46	94%	36	78%	10	22%	Ref		
> 15yrs	222	179	161	90%	119	74%	42	26%	1.27	0.56–3.20	0.55
**Local clinical Features**											
LAP	209	167	152	91%	110	72%	42	28%	Ref		
LAP + Abscess/ sinus	19	16	13	81%	11	85%	2	15%	0.48	0.05–2.30	0.34
**Duration of illness**											
3 months or shorter	90	86	75	87%	52	69%	23	31%	Ref		
Longer than 3 months	77	76	72	95%	53	74%	19	26%	0.81	0.37–1.76	0.56
**Culture Results**											
Positive	137	109	100	92%	73	73%	27	27%	Ref		
Negative	89	74	65	88%	48	74%	17	26%	0.96	0.44–2.1	0.90
**Xpert Results**											
MTB-Detected	124	96	86	90%	64	74%	22	26%	Ref		
MTB-Not detected	99	84	77	92%	55	71%	22	29%	1.16	0.55–2.46	0.66
Bacteriology confirmed* LNTB											
HP Suggestive of TB	158	128	115	90%	88	77%	27	23%	Ref		
HP Not suggestive of TB	20	9	9	100%	4	44%	5	56%	4.07	0.80–21.80	0.034
**Histopathology suggestive of LNTB**											
B. Positive *	158	128	115	90%	88	77%	27	23%	Ref		
B.Negative	50	46	41	89%	29	71%	12	29%	1.35	0.55–3.2	0.46
**Rifampicin sensitive LNTB**											
Isoniazid Sensitive	109	92	84	91%	62	74%	22	26%	Ref		
Isoniazid Resistant	6	5	5	100%	2	40%	3	60%	4.23	0.44–52.7	0.10

LAP: Lymphadenopathy, MTB: *Mycobacterium tuberculosis*,

*Bacteriologically confirmed/B.Positive: Positive on Xpert and/or culture, B.Negative: MTB not detected by Xpert and culture.

## Discussion

LNTB is the second most prevalent manifestation of EPTB in Pakistan^27^. We studied the performance of the histological examination against culture as the gold standard compared to the rapid molecular assay (Xpert) to evaluate the added value of histopathology in the diagnosis of LNTB. People presumed to have LNTB who had no history of previous TB treatment were enrolled. Among presumptive LNTB patients, 390 had interpretable results for both bacteriological and histological examinations. LNTB was diagnosed in 228, including 69% (158) by both bacteriology and histopathology, 9% (20) by bacteriology, and 22% (50) by histopathology suggestive of TB.

In our study, three diagnostic tools were used: histology, Xpert, and culture. Measured by positive culture prevalence of TB was 35% compared to the median prevalence of 30% for 484 LN tissues tested in 10 studies^17^. In our cohort, using CRS, TB was diagnosed in 58.4% (228); among LNTB patients diagnosed, only one patient was HIV reactive, and 4.8% (11) AFB positive. Using the same diagnostic approaches, a higher proportion of TB cases were diagnosed among people investigated in other studies^28,29^. In a study from Ethiopia, LNTB was diagnosed in 80% of patients examined, and among LNTB cases, 9% were HIV positive, and 37% were AFB positive^28^. Similarly, in another study from Pakistan, LNTB was diagnosed in 74% of the people investigated, and 9%of the LNTB patients had a history of previous treatment, with 15% AFB positive^29^. As all three studies were conducted in tertiary care centers, there is a possibility that highly selected patients were being referred to these centers. In addition, a high HIV prevalence and history of previous TB treatment contributed to an even higher prevalence of TB in the study population.

The Xpert MTB/RIF performance in the diagnosis of EPTB in different studies has been evaluated in systematic reviews and meta-analyses^10–13,16,17^. We reported the Se, Sp, PPV, and NPV of Xpert against the culture of 62.4%, 83.3%, 66.9%, and 80.4%, respectively. Systematic review of eleven similar studies (n = 786 LN specimens) reported a pooled Se, Sp, PPV, NPV of 82.4%, 80.3%, 31.6% and 97.6%^17^. We reported a lower sensitivity and NPV and higher specificity and PPV than pooled performance. However, our study results were within the range for Se (50–100%) and Sp (0–100%) reported by individual studies. The lower sensitivity in our cohort was likely due to the exclusion of retreatment TB cases and low HIV prevalence, with the possibility that a higher proportion of study participants had a bacillary load in LN much below the detection limit of microscopy. The smear results in our cohort correlated well with the Xpert cycle threshold (Ct) value. With a Ct value of < 21 as the cutoff for AFB smear-positive, in our cohort, MTB detected by Xpert in 113 AFB smear-negative LN specimens, was low (Ct 22–28) or very low (Ct > 28) in quantity^30^. Despite fewer AFB positives specimens, compared to another study from Pakistan, we reported similar sensitivity (62 vs. 65) and specificity (83 vs. 80%) with a higher PPV (67 vs. 54%), most likely due to better performance of culture.

In our cohort, the Xpert improved bacteriological diagnoses of LNTB by eleven folds compared to microscopy (124 MTB + ve vs.11AFB + ve) but still could diagnose less than 60% of all TB cases. Xpert Ultra is expected to improve sensitivity in such paucibacillary specimens^31,32^. Ultra is reported to have higher sensitivity and lower specificity than Xpert, but the certainty of the evidence is low as the number of studies and patients tested are small^17^.

Compared to other EPTB specimens, a higher heterogeneity in Xpert performance is reported in lymph node tissues^16,17^. Xpert positive results, which are culture-negative, are interpreted as false positive, and low specificity and PPV are often attributed to previous TB treatment^33^. For lymph node TB, concerns are raised about the accuracy of the culture results used as the reference standard^16,17^. To understand false-positive Xpert results in our cohort of new LNTB patients, we analyzed the performance of Xpert stratified by duration of illness. Overall performance was better with significantly higher Se (*P* = 0.012) and PPV (*P* = 0.028) in patients who presented early (Se-76%, PPV-82%) compared to those who presented after a delay of more than 3 month (Se = 52%, PPV = 61%). The host immune responses playing a role in the containment of the disease and the viability of MTB in patients ill for a longer period is the most likely explanation for the detection of MTB-DNA by Xpert, but failure to grow in culture. Future studies in pulmonary and extrapulmonary TB patients are needed to better understand this phenomenon. Besides delays in seeking treatment, other factors that may affect the culture results include variation in the quality and quantity of LN specimens processed for culture^16,17^.

In our study cohort, histology consistent with TB was reported in 208, and necrosis was present in 98% of the LN tissue examined. Compared to our findings, necrosis was reported in 84–85% of histologically diagnosed TB cases in studies from Morocco and Peru^34,35^. In our study*, M. Tuberculosis* infection was confirmed in 75% by culture and/or Xpert, consistent with the results of the other two studies^34,35^. In contrast, four cases with non-necrotizing granuloma were all bacteriologically confirmed in our cohort. It has been reported that a significant proportion of necrotizing granulomas appear infectious with no obvious infectious etiology^36^. We studied treatment response and outcomes in LNTB patients with histopathology suggestive of TB; no difference was reported in treatment outcomes and duration between positive and negative bacteriology cases. In high TB and low HIV prevalent settings like Pakistan, with a high pre-test probability of TB, diagnosis can be made with certainty in tissues showing granulomatous inflammation even in the absence of positive bacteriology.

Among 178 bacteriological confirmed TB cases in our study cohort, histopathology was suggestive of TB in 89% (n = 158), consistent with the results of studies conducted in low HIV prevalent settings^29,34^ but was higher compared to 79% in a study conducted in high HIV setting^35^. The human TB granuloma is the product of a robust cellular immune response to bacterial components. A diminished capacity to mount a CD4^+^ T-cell response correlates with a reduced granuloma-forming capacity^37^. Tuberculosis without tubercles has been reported in HIV-positive patients^38^. Among 20 LNTB cases in our study cohort, HIV status was known for six non-reactive patients. In Pakistan, HIV prevalence is low, but undernourishment is estimated to contribute to 20% of all TB cases^1^. It is plausible that underlying malnutrition was responsible for diminished CD4 + T-cell activity and the absence of granulomatous immune response in these cases. We studied the clinical response to TB treatment among B + ve LNTB patients; a significantly delayed response was observed in patients with non-TB specific morphology (OR:4.07), suggesting weak immune responses^37^. Other reasons that also need consideration for missing granulomatous inflammation in tissue sections include an error made in selecting tissue for histology sections or examining a single section, which is not a true representative of the diseased tissue^5,39^.

In our cohort, the two rifampicin-resistant cases were not detected by Xpert, We also studied the treatment outcome of drug-resistant LNTB patients. Of two rifampicin-resistant patients, one was successfully treated with six-month standard FL-ATT, with a possibility that excision biopsy completed removed diseased lymph nodes, consistent with published evidence of the role of surgery in improving treatment outcomes of LNTB patients^40^. On the contrary, the delayed clinical response was reported in 3/5 INH-resistant TB patients (OR = 4.23), consistent with unfavourable treatment outcomes reported in INH-resistant PTB patients^41^. Xpert has an advantage over histopathology in detecting rifampicin resistance; However, in our cohort, drug resistance, including rifampicin resistance, was diagnosed by phenotypic DST. Culture will still be required in cases where Xpert fails to detect TB, drug resistance TB, or non-tuberculous mycobacteria need to be investigated.

The main shortcomings of our study are; first, it was a single-center study conducted in a specialized tertiary care setting; secondly, only excision biopsy specimens were tested. Therefore, our results and conclusion cannot be generalized to all settings and all types of LN specimens. Third, Xpert MTB/RIF was used with lower sensitivity than the next-generation Xpert Ultra. Fourth, the number of patients was small in some of the subgroups analyzed, and confidence intervals were wide, with a risk of imprecision in “Results”. Fifth, clinical, and radiological data were available for less than 80% of LNTB patients, which may have biased the analysis. Lastly, although 10% of the patients had a chest X-ray suggestive of TB, sputum was examined by AFB microscopy only, with the possibility of under-diagnosis of concomitant PTB.

Xpert has the advantage of rapid diagnosis of TB and resistance to rifampicin. Though culture is of value, long reporting time and limited cultural facilities make it a non-viable option for the majority of the patients in high burden countries. In our study cohort, molecular assay diagnosed 54% of the LNTB cases, consistent with findings from other studies^29,34,35^. With reliance only on Xpert, almost half of the TB cases are likely to be missed; Our finding reinforces the ISTC and the need for clinicians to adhere to standards for evaluating possible EPTB and testing of appropriate specimens for microbiology and histopathology examination^19,20^. In situations where the specimen is not tested, or the molecular assay fails to diagnose TB for any reason, the histopathology examination is of value in suggesting the diagnosis of LNTB. Recent advances to diagnose TB in formalin-fixed paraffin-embedded tissue by detecting TB-specific antigens^26,42^ or MTB by Xpert are likely to improve bacteriological diagnosis of TB without the need for a separate specimen for molecular testing^43^.

Adhering to ISTC would remain a challenge, especially for clinicians working outside tertiary care settings in resource-constrained high disease burden countries like Pakistan. There is a need to improve access to diagnostic services for EPTB specimens by capacity development of an intermediate tier by training clinicians on the use of simpler techniques like fine-needle aspiration and laboratory technicians on handling LN aspirate/biopsy specimen and testing EPTB specimens by Xpert, linking intermediated level with laboratories at a higher level and effective mechanism to transport biopsy specimen/cytology smears^2,27^.

## Conclusion

Diagnosis of EPTB is challenging because of the paucibacillary nature of the disease, the performance of all diagnostic tools currently available is not optimal and negative results don’t rule out TB. For patients presumed to have LNTB, the biopsy specimen should be tested for microbiology and histopathology examinations. In countries like Pakistan with high TB and low HIV prevalence, diagnosis of more than 95% of LNTB cases is possible when Xpert testing is combined with histopathology examination, compared to less than 60% by Xpert alone.

## Data Availability

All relevant data is within the manuscript.
